# Characterizing a standardized BioPart for BAG-specific expression in *C. elegans*

**DOI:** 10.17912/micropub.biology.001150

**Published:** 2024-03-12

**Authors:** Xinglin Qian, Sarah AlHarbi, Christian Frøkjær-Jensen

**Affiliations:** 1 Bioscience, BESE, King Abdullah University of Science and Technology (KAUST), Thuwal, 23955-6900, Kingdom of Saudi Arabia

## Abstract

Biological parts (BioParts) are modular and standardized DNA sequences that encode biological functions and contribute to the efficient biological engineering of complex systems. Here, we characterize a short BioPart (P *flp-17* , 300 bp) for bright multicopy and single-copy BAG-specific expression starting from the gastrula stage in hermaphrodite and male *C. elegans* . We have generated standardized P *flp-17* cloning vectors for BAG-specific *gfp* and *mScarlet* expression compatible with extra-chromosomal arrays and for single-copy transgene insertion. The short P *flp-17* promoter is easy to generate by gene synthesis and has been incorporated into our online transgene design tool (www.wormbuilder.org/transgenebuilder ) .

**Figure 1. Expression and Characterization of P flp-17 in BAG Neurons of C. elegans:**
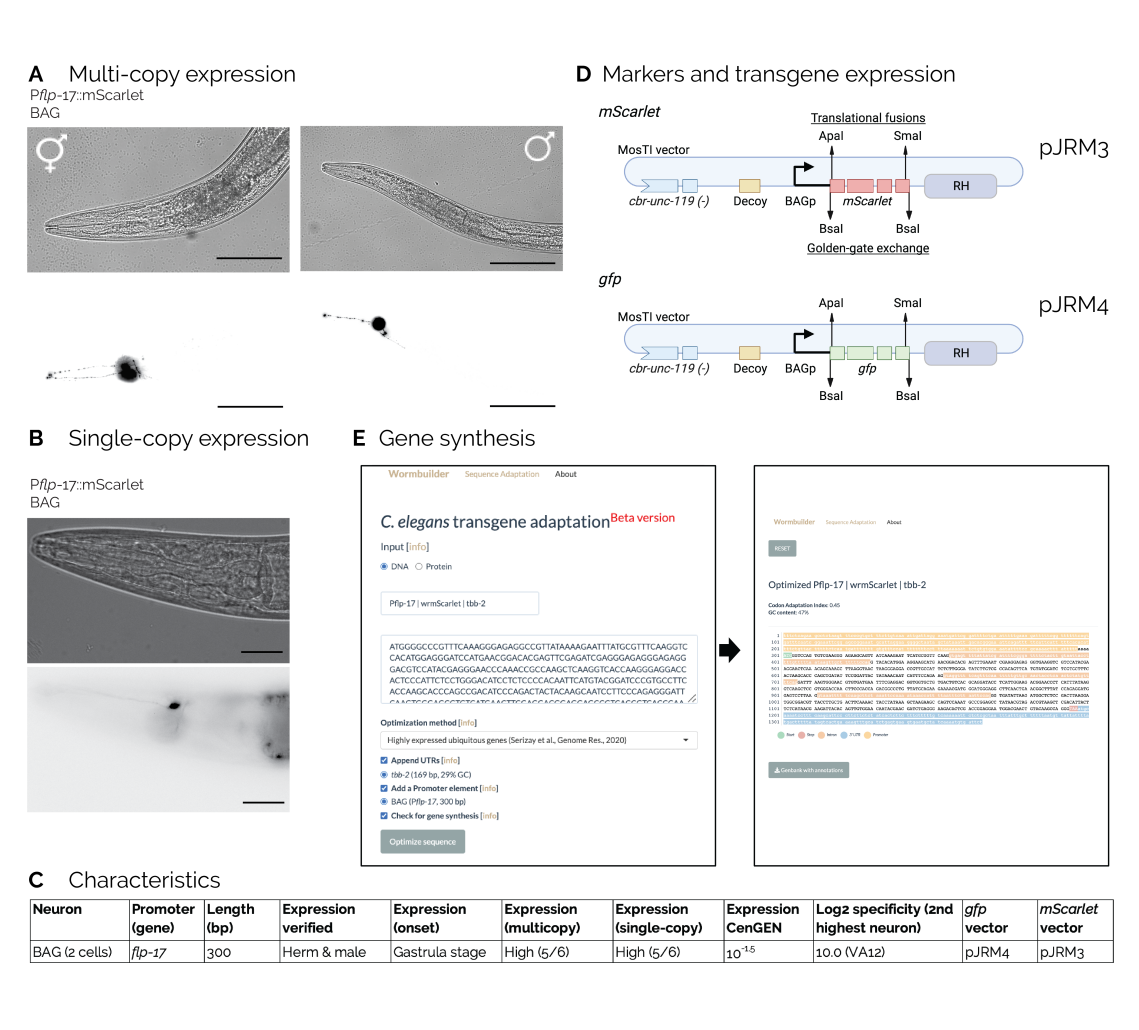
**A** . In hermaphrodites (top panel) and males (bottom panel) a P *flp-17* :: *mScarlet* transgene is expressed exclusively in BAG neurons (20x magnification, scale bars = 100 µm). **B** . Single-copy P *flp-17* :: *mScarlet* expression in a hermaphrodite (20x magnification, scale bar = 20 µm). **C** . *flp-17* promoter characteristics. CenGEN data from https://cengen.shinyapps.io/CengenApp/ and https://cengen.textpressolab.com/. **D** . Schematic of standard *mScarlet* and *gfp* vectors for BAG-specific expression and identification. *Bsa* I restriction sites allow exchanging the fluorescent reporter for a gene of interest by Golden Gate Assembly; *Apa* I (N-terminal) and *Sma* I (C-terminal) allow fluorophore fusions. The vectors are compatible with single-copy insertions (MosTI) and expression from extra-chromosomal arrays. **E** . Illustration of the transgenebuilder workflow. The short 300 bp P *flp-17* was added as a standard promoter in our online tool for optimizing transgene design and gene synthesis (www.wormbuilder.org/transgenebuilder).

## Description

Biological parts (BioParts), such as promoters, 3' UTRs, and protein-coding sequences, refer to DNA sequences that encode well-defined biological functions. Compatible, well-characterized BioParts can benefit the engineering of biological systems by enhancing reliability and reproducibility across laboratories (Shetty et al., 2008) . In *E. coli* and *S. cerevisiae* , substantial effort has been put into developing standardized biological parts and toolkits (Malcı et al., 2022; Moore et al., 2016). In *C. elegans* , the Fire lab vector kits are an early example of the benefits derived from the wide adoption of standardized reagents, and latter examples include comprehensive genome-wide BioPart collections, such as the promoterome (Dupuy et al., 2004) , the ORFeome (Lamesch et al., 2004) , the 3' UTRome (Steber et al., 2019) , and the bacterial RNAi library (Kamath et al., 2003) , which collectively serve as standard reagent platforms and toolkits for the community.

A collection of BioParts encoding promoters that are specific to individual classes of neurons is potentially useful for understanding and controlling the relatively simple *C. elegans* neuronal network consisting of 302 neurons belonging to 118 distinct neuronal classes (White et al., 1986) . For example, neuron-specific expression enables optogenetic recording and manipulation of cellular activity (Suzuki et al., 2003) and genetic screens to identify factors required for neuronal specification (Hobert, 2021) . Many studies have identified individual neuron-specific promoters (e.g., Inada et al., 2006; Sengupta et al., 1996; Yu et al., 1997), and there have been several large-scale efforts to comprehensively identify promoters expressed in single neurons (e.g., Dupuy et al., 2004; Hunt-Newbury et al., 2007). Furthermore, single-cell RNA expression data makes it increasingly feasible to test the expression of promoters from putative cell-specific transcripts (Lorenzo et al., 2020; Nava et al., 2023; Taylor et al., 2021) . We have recently begun an effort to characterize and generate standardized reagents for neuron-specific expression, with a particular emphasis on identifying short promoters that are amenable to gene synthesis (AlHarbi & Frøkjær-Jensen, 2023). The *C. elegans* genome is relatively compact (Cutter et al., 2009) , and several cell-specific neuronal promoter elements are located within the 300 bp of the start codon (e.g., Etchberger et al., 2007; Froehlich & Rajewsky, 2023; Stefanakis et al., 2015; Wenick & Hobert, 2004), suggesting that it may be possible to identify a comprehensive collection of compact promoters. Here, we describe a short promoter for specific expression in BAG neurons, which are involved in sensing oxygen (Zimmer et al., 2009) and carbon dioxide (Bretscher et al., 2011) .

To characterize a 300 bp shortened BAG-specific promoter (Brandt et al., 2012) , P *flp-17* , we generated a fluorescent reporter construct (P *flp-17 ::mScarlet:: tbb-2 3'UTR* ) by gene synthesis. As part of the standardization, we removed *Apa* I, *Sma* I, and *Bsa* I restriction sites to generate standardized reagents, added a standard start sequence ("aaaaATG"), and removed homopolymer runs, which interfere with gene synthesis. To determine expression, we generated transgenic animals with extrachromosomal arrays that carry many copies of the *mScarlet* reporter construct. We also included a piRNA interference (piRNAi) fragment targeting *him-5* (Priyadarshini et al., 2022) to induce males in transgenic animals. We imaged hermaphrodite and male transgenic worms and observed fluorescence starting at the gastrula stage with highly specific red fluorescence in two bilaterally symmetric neurons at the L4 stage that are consistent with the position and morphology of BAG neurons ( **Figure 1A** ). Expression was also readily visible from a single-copy transgene inserted by modular safe-harbor transgene insertion (MosTI) (El Mouridi et al., 2022) ( **Figure 1B** ), demonstrating that the short P *flp* -17 is relatively strong. We have developed a semi-quantitative visual score for expression strength (AlHarbi & Frøkjær-Jensen, 2023) (see methods), and the multicopy arrays scored 5 out of 6, and the single-copy insertion also scored 5 out of 6. The characteristics of the *flp-17* promoter are summarized in **Figure 1C** .

To enable easy use of the P *flp-17* BioPart we have generated standardized expression vectors containing red ( *mScarlet* , pJRM3) and green ( *gfp* , pJRM4) fluorophores in a plasmid backbone that is compatible with single-copy insertion and multi-copy extra-chromosomal arrays ( **Figure 1D** ). The plasmids include restriction sites for N-terminal ( *Apa* I) and C-terminal ( *Sma* I) transgene fusions, sites for fluorophore exchange ( *Bsa* I), and a 5' decoy sequence to diminish misexpression (A. Fire, Fire lab kit). The plasmid design is identical to vectors for PVQ-specific expression (AlHarbi & Frøkjær-Jensen, 2023), and the plasmids are available from Addgene. Finally, the short P *flp-17* promoter is amenable to gene synthesis, and we have included the promoter in an online transgene design app (www.wormbuilder.org/transgenebuilder) ( **Figure 1E** ).

In conclusion, our ongoing efforts aim to develop a comprehensive collection of promoter BioParts for neuron-specific transgene expression. These design principles and reagents represent the initial steps towards establishing such a system. We aim to generate and characterize a complete collection of neuron-specific BioParts to advance our understanding of the nervous system and potentially encode novel circuits.

## Methods

**Molecular biology**

We generated non-clonal synthetic transgenes by gene synthesis (Twist Bioscience, CA, USA) and clonal plasmids by Golden-Gate cloning (Engler et al., 2008) using *Esp* 3I (New England Biolabs). We validated clonal plasmids by restriction digest. We shortened the *flp-17* promoter tested by Lorenzo et al., (2020) to 300 base pairs and removed *Bsa* I, *Esp* 3I, *Apa* I, *Sma* I, and *Eco* RV, as well as homopolymer runs. We changed the last four basepairs of the promoter to the consensus start site (aaaaATG).

**Promoter strength quantification and expression onset determination**

We quantified the fluorescence intensity of *mScarlet* reporter constructs using a scheme first described by Alharbi and Frøkjær-Jensen (2023). Transgenic animals with stable extra-chromosomal arrays under a dissection stereo microscope (ZEISS, Olympus SZX2-FOF) or an upright compound microscope (LEICA DM2500 LED), objectives 1x and 10x using an mTomato filter set and an LED light (X-Cite XYLIS, XT720L) with 40x oil immersion objective, a Rhodamine filter set (LEICA 11504205), and a mercury metal halide bulb (LEICA EL6000). We quantified fluorescence intensity visually in ten L4 animals at different magnifications. We performed the quantification by eye and scored on a scale from 1 to 6, with 1 being the dimmest and 6 being the brightest. We used the following scoring criteria. **Dissection microscope** . Score = 6: fluorescence visible with 1x objective and zoom = 1 (lowest). Score = 5: fluorescence visible with 1x objective and zoom = 8 (highest). Score = 4: fluorescence visible with 10x objective and zoom = 1. Score = 3: fluorescence visible with 10x objective and zoom = 8. **Compound microscope** . Score = 2: fluorescence visible with 20x air objective. Score = 1: fluorescence visible with 40x oil immersion objective. Score = 0: fluorescence not visible at 40x oil immersion objective. We screened 15 embryos from transgenic animals at various stages (gastrula, comma, 1.5-fold, 2-fold, and 3-fold) on NGM plates for the first visible expression.

**Microscopy** We anesthetized transgenic animals on 2% agarose pads with a 50 mM sodium-azide M9 solution. Extra-chromosomal array animals were imaged with a Leica THUNDER Imaging System, equipped with a 20× oil immersion objective, and captured four image stacks—two for hermaphrodites and two for males. The images show a maximum intensity projection generated with the Leica LAS X software. Animals with single-copy insertions were imaged on a LEICA DM2500 LED using a 42x oil immersion objective.

**Transgenic animals**

To generate extrachromosomal arrays animals, we injected the injection mix composed of 10 ng·µL ^-1^ non-clonal P *flp-17 ::mScarlet:: tbb-2* 3' UTR dsDNA fragment, 10 ng·µL ^-1^ pCFJ108 ( *unc-119* rescue, linearized by *Apa* LI), 10 ng·µL ^-1^ pCFJ782 (HygroR, linearized by *Eco* RV), 10 ng·µL ^-1^ pMNK54 (piRNAi *him-5* , linearized by *Apa* LI), and 60 ng·µL ^-1^ GeneRuler 1 kb plus DNA ladder (ThermoFisher SM1331) for a final concentration of 100 ng·µL ^-1^ , into CFJ42 animals (MosTI II, *unc-119* ( *ed3* ) III). We kept injected animals at 25℃ and added hygromycin to the plates on day 3. We identified transgenic animals with extrachromosomal arrays based on *unc-119* rescue and antibiotic resistance.

Single-copy transgenes with pJRM3 (P *flp-17* :: *mScarlet* :: *tbb-2* 3' UTR) were inserted by MosTI into a landing site on Chr II ( CFJ42 , *ttTi5605* site) following standard protocols (El Mouridi et al., 2022) . The injection mix was comprised of 20 ng·µL ^-1^ pJRM3 (P *flp-17* :: *mScarlet* ) MosTI targeting vector, 15 ng·µL ^-1^ pSEM318 (MosTI sgRNA, linearized by *Nde* I), 10 ng·µL ^-1^ pSEM231 (P *mlc-1* :: *gfp* , co-injection marker, linearized by *Nde* I) (El Mouridi et al., 2020) , 15 ng·µL ^-1^ pCFJ782 (hygromycin resistance, linearized by *Eco* RI) (Radman et al., 2013) , 25 ng·µL ^-1^ pMDJ231 (heat-shock Cas9, linearized by *Apa* LI), and 15 ng·µL ^-1^ GeneRuler 1 kb plus DNA ladder (ThermoFisher SM1331) for a final concentration of 100 ng·µL ^-1^ . We kept injected animals at 25℃ and added hygromycin to the plates on day 3. When the bacterial lawn was nearly exhausted, we heat-shocked the plates with transgenic animals in a 30℃ air incubator for 20 hours and kept animals at 20℃ for four days. We identified transgenic animals with a single-copy insertion four days after the heat shock based on *unc-119* rescue and the absence of green co-injection markers.

**Software**

We performed *in silico* design with “A plasmid Editor” (ApE) (Davis & Jorgensen, 2022) , and generated the figure with Adobe Illustrator (v28.2).

A frozen version of the transgenebuilder software was described in Alharbi & Frøkjær-Jensen (2023) and is available at https://doi.org/10.22002/qs7eh-g0669.

## Reagents

**Plasmids**

pJRM3 - P *flp-17 ::mScarlet:: tbb-2* 3' UTR (MosTI compatible) (Addgene #204616)

pJRM4 - P *flp-17 ::gfp:: tbb-2* 3' UTR (MosTI compatible) (Addgene #204617)

pMNK54 - *him-5* piRNAi fragment (Addgene #159818) (Priyadarshini et al., 2022)

pCFJ108 - *cbr- unc-119* (Addgene #200367)

pSEM231 - *mlc-1* p:: *gfp* :: *cbr- tbb-2* (Addgene # 159897) (El Mouridi et al., 2020)

pSEM318 - *rpr-1* p::sgRNA targeting the *ttTi5605* location Chr. II (Addgene # 159822)

pMDJ231 – *hsp-16.41* p:: *cas9* :: *gpd-2* :: *tagRFP-t* :: *smu* -1 (Addgene # 191382)

pCFJ782 - P *rps-0 ::hygromycinR:: rps-27* 3' UTR (Addgene #190933) (Radman et al., 2013) .

**Strains**

N2 – (wildtype)

CFJ42 – *kstSi42* [ *unc-119* (p1, spc2)(-)] II; *unc-119* ( ed3 ) III (available from CGC)

**Promoter sequence**

**Table:** 

WormBase ID	Gene Name	Promoter Sequence (modifications in upper case, consensus start site in bold)
WBGene00001460	*flp-17*	tttctcagaagcctctaagtttcccgtgctttcttgtcaaattgattaggaaatgattcggattttctgaatttttgaaa gatttttcggttttttcagtgatttcaatcggaaattcggagccggaaatgcattaggaaggggctaatagctataa attgacacgggaaattcagatttttcattcatttttcacacattttctgttactttttctcaatgatttttttgtgtttccat tttttttcctttaaaaaaattctgtgtggaaatattttccgCaaaactttatttttaaaaagagacc **aaaaATG**
